# Use of Melinex film for flat embedding tissue sections in LR White

**DOI:** 10.1111/jmi.13359

**Published:** 2024-09-16

**Authors:** C. J. von Ruhland

**Affiliations:** ^1^ Electron and Light Microscopy Facility Central Biotechnology Services School of Medicine Cardiff University Cardiff UK

**Keywords:** acrylic resin, flat embedding, LR White, Melinex film

## Abstract

Tissue slices can undergo distortions during processing into resin for light and electron microscopy as a result of differential shrinkage of the various tissue components, and this may necessitate removal of a considerable amount of material from the final resin‐embedded tissue block to ensure production of complete sections of the sample. To mitigate this problem, a number of techniques have been devised that ensure the sample is held flat during the final curing/polymerisation of the resin. For embedding in acrylic resins, oxygen must be excluded as it inhibits polymerisation, and methods devised for epoxy resin embedding are generally unsuitable. The method describes the preparation and use of air‐tight flat‐embedding chambers prepared from Melinex film and provides an inexpensive, technically simpler, and versatile alternative to chambers formed from either Thermanox coverslips or Aclar films that have previously been advocated for such purposes.

**Lay description**: Tissue slices can undergo distortions during processing into resin for light and electron microscopy as a result of differential shrinkage of the various tissue components. Such distortions may necessitate removal of a considerable amount of material to ensure production of complete sections of the sample. For embedding in acrylic resins, oxygen must be excluded as it inhibits polymerisation, and methods devised for epoxy resin flat‐embedding are generally unsuitable. Air‐tight flat‐embedding chambers prepared from either Thermanox coverslips, or a combination of PTFE‐coated glass slides, polycarbonate film gaskets, and Aclar film have been advocated for such purposes. Thermanox coverslips are expensive and limited in size to 22 mm × 60 mm, and the alternative method is technically complicated. Melinex film is commercially available as 210 mm × 297 mm sheets and is approximately 1/20th the price of Thermanox and less than half the price of Aclar film. The method describes the preparation and use of embedding chambers made from Melinex film, glass slides and double‐sided adhesive tape as a technically simpler, inexpensive and versatile alternative to both Thermanox coverslips and the Aclar film method.

## 1 INTRODUCTION

Thick tissue sections, such as those prepared using a vibratome, have the potential to curl or distort as a consequence of differential shrinkage during preparation for resin embedding. Such distortions may necessitate trimming through a considerable depth of the sample before sections of the entire specimen can be cut. In the most extreme cases, this may not even be possible. For epoxy resin embedding, methods have been developed for a number of tissues to ensure that the specimen remains flattened during the final curing process including brain,^1^, ^2^, ^3^ neurones,^4^ muscle,^5^ and tissue cryosections.^6^ Acrylic resins offer a number of advantages over epoxy resins including (1) lower viscosity, facilitating infiltration, (2) the avoidance of post‐fixation with osmium tetroxide, whilst retaining good ultrastructural preservation, allowing a greater variety of histochemical techniques to be employed, (3) tolerance of small quantities of water, permitting partial dehydration and retention of greater tissue reactivity, and (4) immunolabelling without the need for etching,^7^ but oxygen must be excluded to prevent inhibition of polymerisation, and the above methods are generally unsuitable. Air‐tight moulds formed from either Thermanox coverslips,^8^ or a combination of PTFE‐coated glass slides, polycarbonate film gaskets and Aclar,^9^ have been advocated for acrylic resin‐embedding of free‐floating cells or cryofixed plant tissue, respectively. Thermanox coverslips, while not too expensive individually, may be difficult to justify financially if only occasional use is required, as they only seem to be available in minimum pack sizes of 100, or 500 in the case of several suppliers. The latter method, while offering a less expensive alternative, is technically complicated, and a simpler and cheap alternative to both seems desirable.

Melinex films were first advocated for use in electron microscopy as easily separable tissue culture supports for epoxy resin embedding^10^ and are commercially available in small packs at very low cost; less than £30 for 5 sheets measuring 210 mm × 297 mm × 175 µm, which is less than half the price of Aclar film, and approximately 1/20th the price of Thermanox coverslips. Moreover, Thermanox coverslips are only available up to a maximum size of 22 mm × 60 mm. The following is a modification of Vesk et al.’s method^8^ that substitutes Thermanox with Melinex to produce a simple, low cost mould for oxygen‐free, flat embedding of vibratome tissue sections in LR White acrylic resin.

## 2 MATERIALS AND METHODS

Melinex sheets, hard grade LR White acrylic resin and accelerator were purchased from Agar Scientific (Stanstead, Essex, UK). Sellotape double‐sided adhesive tape (product code 15474294) (D/S tape) was supplied by Fisher Scientific (Loughborough, Leicestershire, UK). Sellotape single sided‐adhesive tape (S/S tape) (Product Code: 6.004.457) was purchased from Lyreco (Telford, Shropshire, UK). To illustrate the method, 100 µm, 200 µm and 300 µm thick sections of archival 1% glutaraldehyde perfusion‐fixed rat kidney, liver and duodenum, and 4% formaldehyde + 0.2% glutaraldehyde immersion‐fixed mouse tumour were cut with a Leica VT1200S vibratome (Leica Microsystems, Milton Keynes, Buckinghamshire, UK), fully dehydrated through graded isopropanol (IPA) (10 min each in 50% and 70%, 2×15 min in 100%) and infiltrated with LR White resin (20 min in 50% resin in IPA, 4×20 min in neat resin).

**FIGURE 1:**
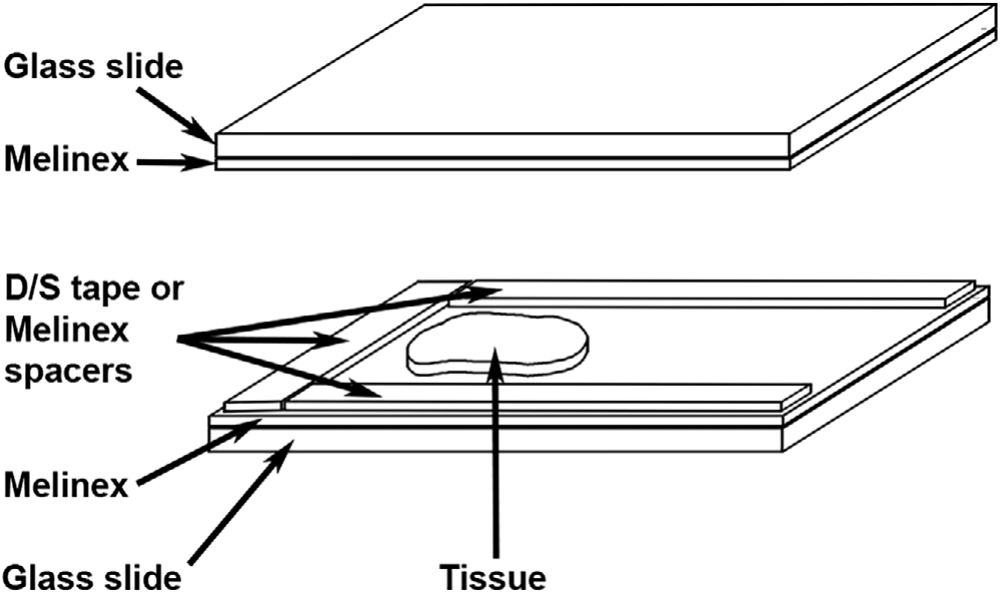
Components of flat‐embedding mould.

**FIGURE 2:**
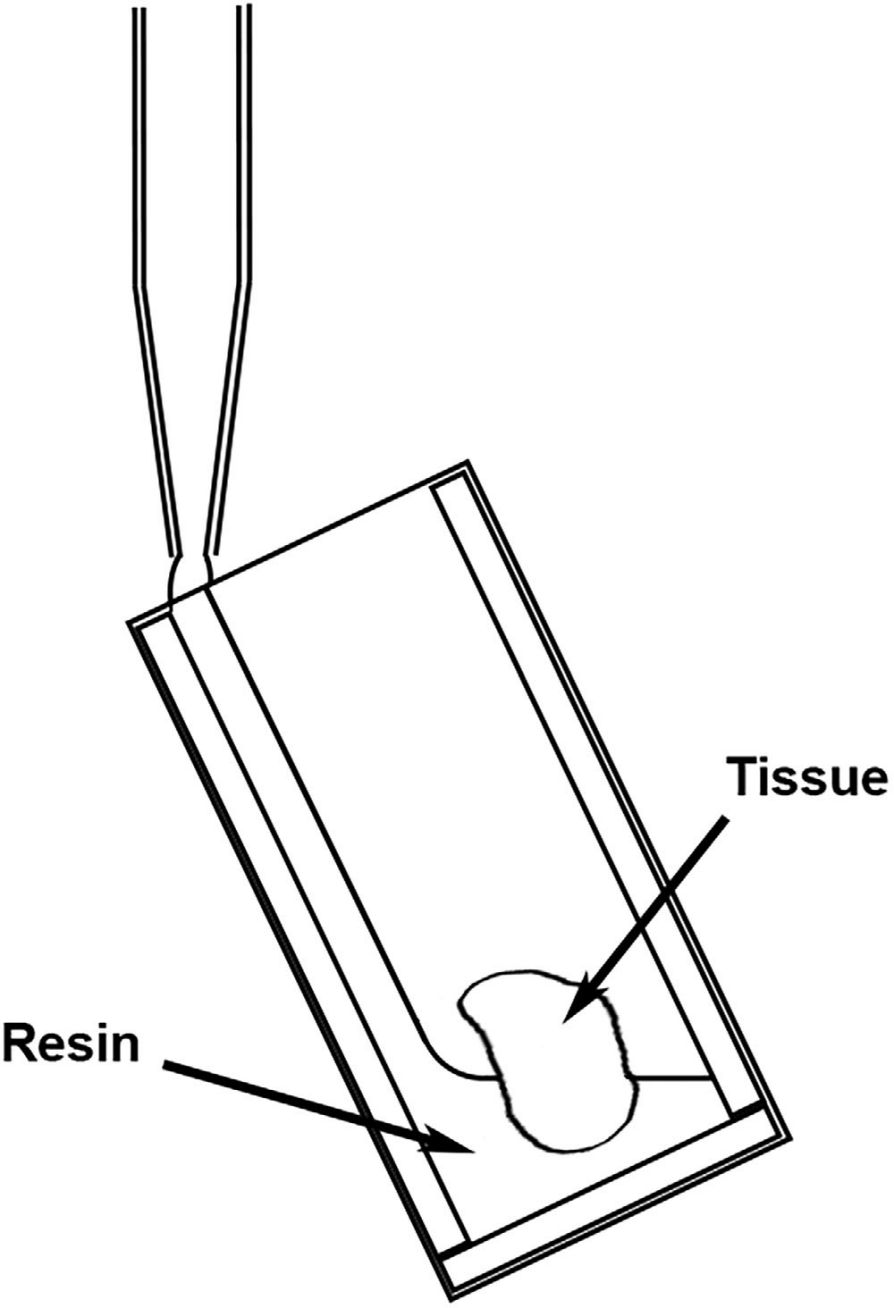
Filling of flat‐embedding mould to exclude air bubbles.

Flat embedding chambers were prepared as follows. 26 mm × 76 mm strips of Melinex film were adhered to glass slides with D/S tape. For embedding 100 µm thick tissue sections, 4–5 mm wide strips of D/S tape were attached around 3 sides of the film‐covered slide to form a gasket. For thicker sections, either multiple layers of D/S tape, or 4–5 mm wide strips of Melinex film, with D/S tape on both sides, were used. A tissue section was placed close to the bottom of the resulting well and a Melinex film‐coated slide placed on top, gently holding the section in place (Figure 1). The resulting mould was sealed with S/S tape, to prevent any leakage of resin during polymerisation that might occur if the joints between the gasket strips were not sufficiently tight, and filled with fresh resin using a fine pipette. To avoid any air becoming trapped during filling, the assembly was tilted to allow the resin to run down one side on the well and fill from the bottom (Figure 2). The assembly was held vertically in a Coplin jar and placed overnight in an oven at 50°C. Once polymerisation was complete, unpolymerised resin near the well opening was drained off by inserting a piece of filter paper into the top of the well, and the assembly separated by removing the sealing tape, inserting a scalpel blade between the Melinex film and the spacers, and twisting to force one of the Melinex film‐coated slides off. Any remaining unpolymerised resin was wiped off the sample with IPA.

The resulting resin‐embedded samples were adhered to blank LR White blocks with a 1:20 mixture of accelerator and LR White, surplus overhanging resin trimmed away with a scalpel, and 5 µm thick sections cut with a tungsten knife on a Leica 2155 motorised microtome (Leica Microsystems, Milton Keynes, Buckinghamshire, UK). Sections were stained by immersion in 0.02% aqueous toluidine blue for 10 s, thoroughly washed in water, air dried and mounted with Gurr's neutral mountant. Sections were examined with an Olympus BX51 research light microscope (Olympus Optical Co. (UK) Ltd, London, UK) and digital photomicrographs captured with a Zeiss Axiocam and Axiovision software (Carl Zeiss Vision GmbH, Hallbergmoos, Germany).

## 3 RESULTS

Unpolymerised resin formed a layer on the upper edge to a depth of approximately 5 mm. Placing the tissue section at the lower end of the well ensured embedding in fully polymerised resin.

**FIGURE 3:**
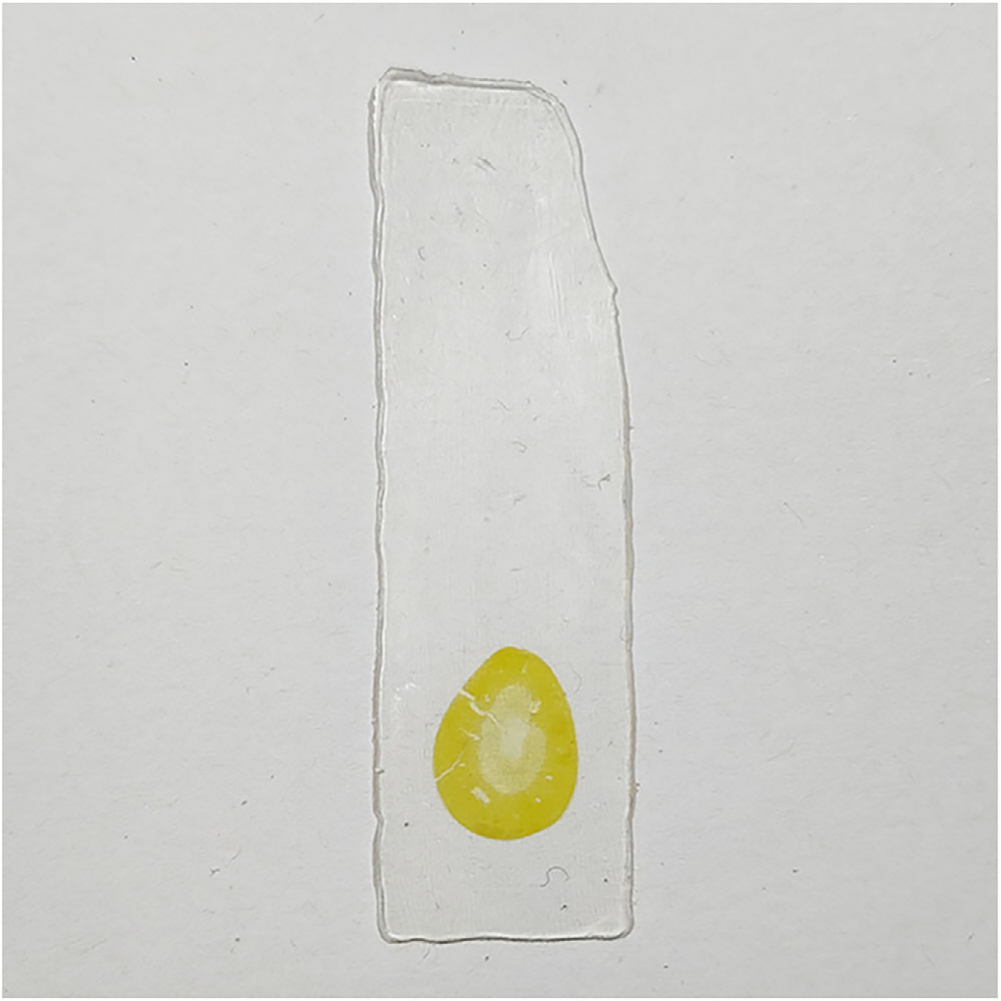
Flat‐embedded 200 µm thick kidney section.

**FIGURE 4:**
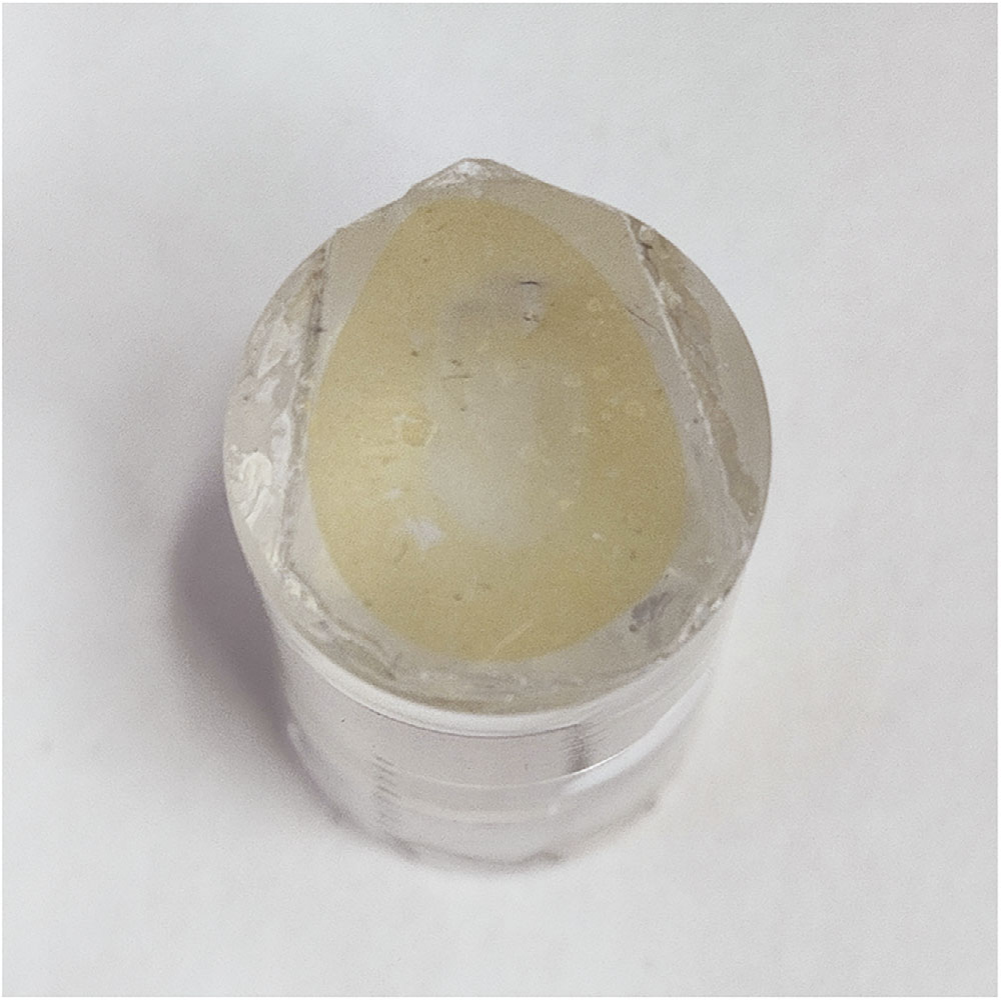
Flat‐embedded 100 µm thick kidney section adhered to blank resin block to facilitate removal from the embedding mould.

Upon separating the assembly, the resin‐embedded sample remained adhered to one of the Melinex‐coated slides. Thicker resin‐embedded sections (200 µm thick or more) were easily separated by sliding a scalpel blade between the resin and the Melinex film to produce a thin sheet of resin‐embedded tissue (Figure 3). Thin (100 µm thick) sections that were embedded using only D/S tape as a spacer were too thin and brittle to be removed in this manner and required pre‐bonding to the flat surface of a blank resin block using either cyanoacrylate adhesive or a 1:20 mixture of accelerator and LR White to provide mechanical support during separation from the Melinex‐coated slide (Figure 4).

**FIGURE 5:**
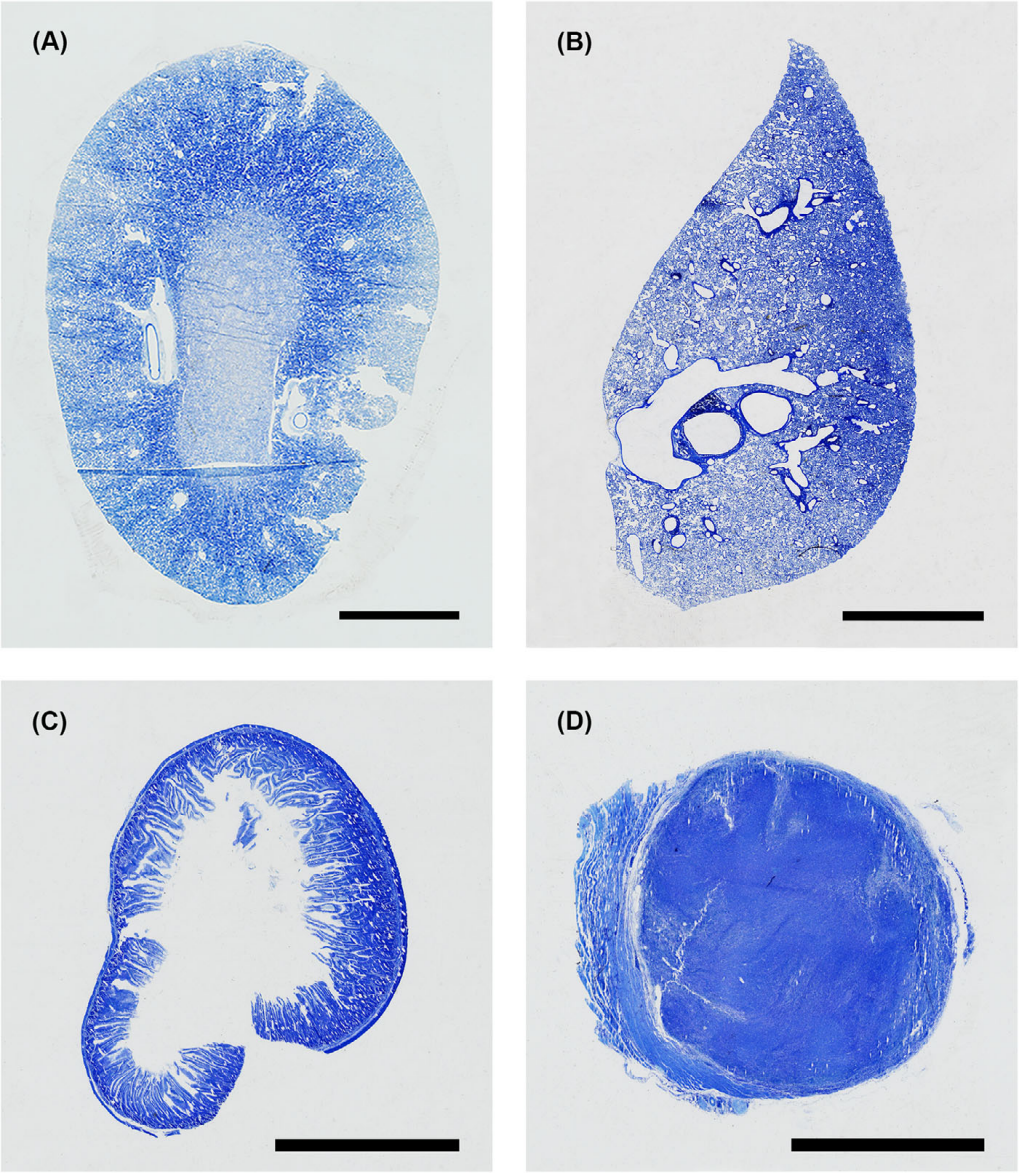
Light micrographs of toluidine blue‐stained 5 µm thick sections of rat kidney (A), liver (B), duodenum (C), and mouse tumour (D). Scale bars 5 mm.

Resin‐embedded samples remained firmly attached to the blank stubs during subsequent sectioning, and complete tissue sections could quickly be achieved with minimal trimming into the block (Figure 5).

## 4 DISCUSSION

The technique, described above, provides a cheap and simple method for flat‐embedding vibratome sections of tissue in acrylic resin and offers a number of advantages over previously described methods. Melinex film is approximately 1/20th the price of Thermanox coverslips used in Vesk et al.'s method^8^ and its availability in A4 sheets allows much larger tissue sections than illustrated here to be embedded by simply selecting appropriately sized glass slides or sheets thus overcoming the size limitations imposed by Thermanox coverslips, which are only available up to a maximum size of 22 mm × 60 mm. The method of Palmieri and Kiss,^9^ requires spraying slides twice with PTFE (presumably in a fume cabinet) followed by polishing to facilitate removal of the polymerised resin‐embedded sample, the use of silicone adhesive (requiring 24 h curing at RT or 1 h at 60°C) to attach the polycarbonate gasket, and Aclar film. In contrast, the technique described here require only 2 components and can be easily and quickly assembled on the open bench. In addition, Melinex film is less than half the price of Aclar. Both previously described methods employ a complete, 4‐sided gasket to form a well for the sample and resin and these must necessarily be overfilled to ensure that no air bubbles are trapped when the final layer of the assemblies are put in place, resulting in spillage of resin, whereas the 3‐sided gasket used in the technique proposed here avoids this problem.

Four types of animal tissue were chosen to illustrate the applicability of the technique, but equally good results should be achievable with other tissue types such as plant, as used by Palmieri and Kiss^9^ and readers are encourage to experiment.

D/S tape is approximately 100 µm thick, and Melinex film from microscopy suppliers is 175 µm thick, thus almost any thickness of tissue can be accommodated by combining layers of the two materials into spacers as appropriate. A complete, 3‐sided gasket could also be easily prepared should there be any concerns regarding the patency of seals where the strips meet.

With a little ingenuity, the method described above should also be suitable for acrylic resin‐embedding of large areas of cultured cells grown on Melinex films, allowing all the cells to be embedded in a single sheet of resin. Pieces can then be cut out and mounted onto blank resin blocks, as described above, for subsequent sectioning and should provide a useful alternative to other flat embedding methods such as that described by Steiner et al.^11^

Since Melinex film was originally introduced as an easily separable support for flat‐embedding cultured cells in epoxy resin,^10^ the technique described here should be equally applicable for epoxy resin‐embedding of tissue sections, as long as low viscosity formulations are used.
